# Comprehensive assessment of metabolic syndrome among rural Bangladeshi women

**DOI:** 10.1186/1471-2458-12-49

**Published:** 2012-01-19

**Authors:** Subrina Jesmin, Md Reazul Islam, A M Shahidul Islam, Md Sohag Mia, Sayeeda Nusrat Sultana, Sohel Zaedi, Naoto Yamaguchi, Yoshio Iwashima, Michiaki Hiroe, Tetsu Watanabe

**Affiliations:** 1 National Center for Global Health and Medicine, 1-21-1 Toyama, Shinjuku-ku, Tokyo 162-8655, Japan; 2 Health and Disease Research Center for Rural Peoples, Ena Arista, Flat # B-3, House # 802, Road # 3, Baitul Aman Housing Society, Adabor, Dhaka 1207, Bangladesh; 3 Center for Medical Sciences, Ibaraki Prefectural University of Health Sciences, Ami-machi, Inashiki-gun, Ibaraki 300-0394, Japan; 4 Division of Hypertension and Nephrology, National Cardiovascular Center of Japan, 5-7-1, Fujishirodai, Suita City, Osaka, Japan; 5 Department of public Health, Tokai University Graduate School of Medicine, Isehara, Japan

**Keywords:** Metabolic syndrome X, Asia, Western, Bangladesh, Rural populations, Women

## Abstract

**Background:**

Metabolic syndrome (MS), defined as a constellation of cardiovascular disease (CVD) risk factors, is one of the fastest growing public health burdens in the Asia-Pacific region. This trend is despite the fact that people in this region are no more overweight than Europeans and Americans. Unfortunately, in South Asia, MS screening has only been performed in a few countries other than Bangladesh. Therefore the present study is designed to conduct a comprehensive screening of MS in Bangladeshi rural women, which includes estimation of prevalence and assessment of risk factor.

**Methods:**

A total of 1535 rural Bangladesh women aged ≥ 15 years were studied using a population based cross-sectional survey. The prevalence of MS was estimated using NCEP ATP III, modified NCEP ATP III and IDF criteria.

**Results:**

The prevalence rates of MS were 25.60% (NCEP ATP III), 36.68% (modified NCEP ATP III), and 19.80% (IDF), as revealed by the present study. Furthermore, based on the NCEP ATP III criteria, 11.60% of the subjects were found to have excess waist circumference; 29.12% had elevated blood pressure, 30.42% had elevated fasting plasma glucose level, 85.47% had low HDL values and 26.91% had increased triglyceride values. Low plasma HDL level was found to be the most common abnormality in the target population and elevated waist circumference was the least frequent component.

**Conclusions:**

The present study reveals a high prevalence of MS and its associated risk factors in rural Bangladeshi women. These findings are important in that they provide insights that will be helpful in formulating effective public health policy, notably the development of future health prevention strategies in Bangladesh.

## Background

Metabolic syndrome (MS) is described as a cluster of abnormalities that confers an increased risk of developing atherosclerotic cardiovascular diseases (CVD) and also type 2 diabetes mellitus (T2DM). In fact, MS is now considered as a global epidemic [1], with current estimates about 20-30% of the adult population worldwide is affected [2]. Of interest to the current study is the fact that the prevalence of these disorders (MS and CVD) among South Asians [3], a community that represents one-fifth of the global population [4], is on the rise. This development imposes a significant health burden to the subcontinent that has limited resources. The exact cause of this health trend is currently not clear. Nonetheless, several factors are believed to be potential etiological factors in the development of MS, including increased urbanization, economic growth, irregular meal times, and lifestyle change and adoption of western diet [5]. Unfortunately, millions of people in South Asia are facing a double health burden [2] i.e., the impact of poverty-related diseases (associated with infections and nutrition) is being exacerbated by the increasing load of chronic non-communicable diseases [6]. To date there has been very few studies conducted on rural women in Bangladesh. Because of our earlier preliminary data on this population were alarming, we decided to undertake a more detailed study to investigate the metabolic syndrome in rural Bangladeshi population, who are mostly poor and physically active. We found a higher prevalence of diabetes among Bangladeshi women, surprisingly, even in those living in rural regions [7].

The purpose of the present study was; therefore, to evaluate the prevalence of MS, identify the set of signature risk factors of MS and CVD that are unique to South Asia. Further we will examine the correlation of MS and CVD to the socio-economic and environmental conditions, particularly in Bangladesh rural women.

## Methods

### Participants

In a community based cross-sectional study of rural women, a total of 1535 participants aged ≥ 15 years were selected using stratified multistage random sampling. This sample size was sufficient enough to test the research hypothesis at 5% level of significance with a statistical power of 90% (β = 0.90). The study uses the World Health Organization's STEPS approach (modified), which entails a stepwise collection of the risk factor data based on standardized questionnaires covering demographic characteristics, somatic illnesses, somatic and mental symptoms, medications, life style, and health-related behavior (step 1), basic physical measures (step 2) and basic biochemical investigations, such as levels of blood glucose and cholesterol (step 3). All participants gave informed consent.

The study was carried out in six village communities in Gabindagonj upazila (sub-district) of Gaibandha district. This is a district in Northern Bangladesh (currently in Rangpur division, the poorest region of the country, Bangladesh); the respondents were selected randomly after selecting the division, district, and villages. Gaibandha district was established in 1984. The district consists of 7 upazilas, 3 municipalities, 27 wards, 82 union parishads, 1101 mouzas, 56 mahallas and 1244 villages. 7 upazilas/thanas are Fulchhari, Gaibandhasadar, Gobindaganj, Palashbari, Sadullapur, Saghata, and Sundarganj. The three municipalities are Gaibandha Sadar, Gobindaganj and Sundarganj.

The demographic profiles of the communities are (year 2003): 2,117,959, i.e., males 50.26% and females 49.74%. Average literacy 24.3%, i.e., male 31.6% and female 16.5%. Women were recruited through local announcements (loudspeaker) at community level and by house-to-house visits. After recruitment, participants were interviewed and examined clinically at mobile examination center and their blood samples were collected.

Furthermore, the socio-demographic-economic status and physical activity of the participants was assessed using standard methods. Participants with the following conditions were excluded from the study: pregnancy, any acute illness, on steroidal medications or any other medications likely to cause elevated plasma glucose, suffering from chronic renal or pancreatic diseases, on hormone replacement therapy (HRT) as well as individuals with known illness like ischemic heart disease (IHD), diabetes, and hypertension, and individuals who have lived in Gabindagonj for less than 6 months.

The study was approved by the Ethical Committee of the Health and Disease Research Center of Rural Peoples (HDRCRP), Dhaka, Bangladesh. This study conformed to the principles outlined in the Helsinki Declaration, and all subjects gave their written informed consent before inclusion in the study.

### Anthropometry

The following parameters were measured thrice and the mean was then determined: height (cm), weight (kg) and waist circumference (cm). For measuring height, subjects were requested to stand upright on a stadiometer, with their back against the wall, eyes directed forward, without shoes and with their heels aligned together. Weight was measured to the nearest 100 g using a digital weighing machine that was kept on a firm flat surface and checked with 'known' weights every day. Waist circumference was measured with a non-stretchable tape at the midpoint between the lower border of the rib cage and upper border of the iliac crest. Blood pressure (BP) was measured using the standard mercury manometer and cuff, twice in the right arm in a sitting position, to the nearest 2 mmHg, with the initial reading taken at least 5 min after the subject was made comfortable, and again after an interval of 15 min. The average systolic and diastolic blood pressure were then determined.

### Biochemical analysis

Blood for biochemical analysis was obtained from the participants after 10-12 h of an overnight fast. The serum was immediately separated by centrifugation, and the concentration of triglycerides (TG) [lipoprotein lipase method; Wako Chemicals, Tokyo, Japan], total cholesterol (TC) [Cholesterol E, Wako Pure Chemical Industries, Ltd. Osaka, Japan]and its fractions [low-density lipoproteins (LDL)] and high-density lipoproteins (HDL) [high density lipoprotein (HDL)-cholesterol with the Determiner-L kit (Kyowa Co Ltd, Tokyo, Japan)] were ascertained. Fasting plasma glucose [glucose with the Hexokinase G-6-PDH kit (Wako Pure Chemical Industries Ltd, Osaka, Japan)] and insulin were also measured. LDL-cholesterol was calculated as total cholesterol--HDL-cholesterol--VLDL-cholesterol; VLDL-cholesterol was calculated as 0.456 × total triglyceride concentration expressed in mmol/L (Friedewald).

### Definition of metabolic syndrome

The major components of MS include central obesity, elevated BP, dyslipidemia and impaired glucose metabolism or insulin resistance. According to the NCEP ATP III definition, MS is diagnosed when 3 or more of the following 5 components are present: (1) elevated waist circumference (WC ≥ 88 cm in women), (2) elevated triglyceride (TG ≥ 150 mg/dl, ≥ 1.7 mmol/L), (3) reduced HDL cholesterol (HDL < 50 mg/dl or < 1.29 mmol/L in women), (4) elevated blood pressure (BP ≥ 130/85 mmHg), and (5) elevated fasting plasma glucose (FPG ≥ 110 mg/dl or ≥ 6.1 mmol/L) or pre-existing diabetes mellitus (DM).

In addition to the NCEP ATP III [8] definition, we have used International Diabetes Federation (IDF) [9] and NCEP ATP III criteria modified for Asian subjects (WC ≥ 80 cm in women) as recommended by an American Heart Association-National Heart Lung Blood Institute (AHA/NHLBI) statement [10]. The modified NCEP ATP III criteria were the same as those of the IDF criteria except that the waist circumference criterion was non-obligatory.

### Socioeconomic status and variables

Socioeconomic status (SES) has been identified as an important determinant of health across a broad range of health issues [11,12]. SES is a multidimensional construct that includes education, occupation, income, wealth, and residence. However, the measure of this construct in less-developed countries creates a challenge because adequate data may not be available. Therefore, inclusion of several measures of SES is advantageous because it increases the likelihood of capturing a broader and, subsequently, more informative SES constructs [12]. No previous studies of MS have explicitly examined the effect of modifying SES in the Bangladeshi context. We sought to determine whether selected SES indicator variables modify the association between various measures of MS among 1535 rural Bangladeshi women.

We considered education, family income, land ownership and living housing area as indicators of SES following Argos et al. [12] and Durkin et al. [13]. The baseline questionnaire measured years of education, family income, land ownership and living housing area for all participants.

### Statistical analysis

The statistical analysis was carried out using Statistical Package for Social Sciences (SPSS Inc., Chicago, IL, version 18.0 for Windows). All quantitative variables were estimated using measures of central location (mean, median) and measures of dispersion (standard deviation and standard error). Proportions were compared using Chi square test. Logistic regression analysis was applied to find out independent predictors for MS. All statistical tests were two-sided and performed at a significance level of *p *= 0.05.

## Results

**Figure 1:**
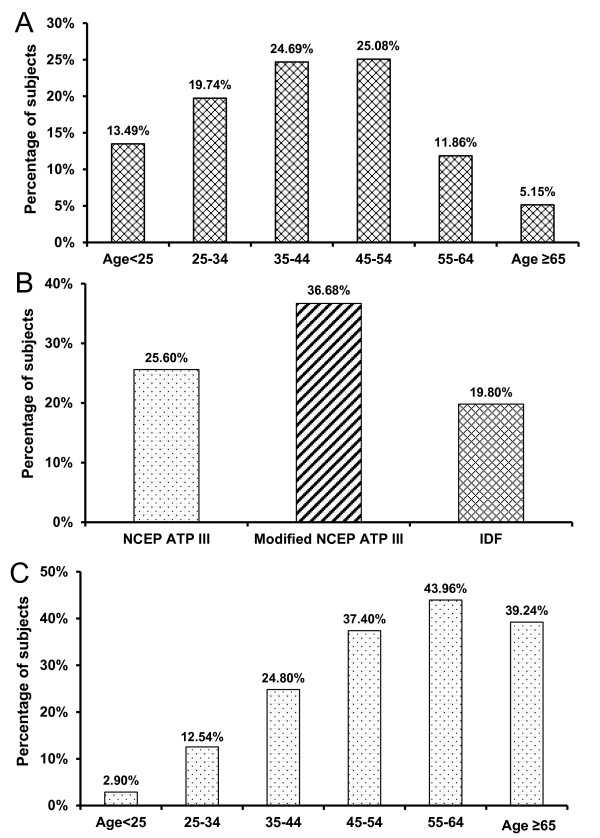
**(A) Age distribution of study subjects (n = 1535), (B) Prevalence of metabolic syndrome of Bangladeshi rural women based on three established criteria (NECP ATP III, Modified NECP ATP III, IDF), (C) Age stratified prevalence of metabolic syndrome of Bangladeshi rural women (NCEP ATP III criteria)**.

The present study is, to date, one of the largest community-based and comprehensive MS surveys done on rural Bangladeshi women. A total of 1,535 females aged between 15 and 85 years were included for analysis in the present study (mean ± SE age of 40.50 ± 0.35 years). Women aged 35 to 54 represented about half of the participants; whereas elderly women aged 65 and above represented only 5.15% (Figure 1A). The age-wise distribution of subjects were 13.49% (< 25 years), 19.74% (25-34 years), 24.69% (35-44 years), 25.08% (45-54 years), 11.86% (55-64 years) and 5.15% (65 years+) (Figure 1A).

### Prevalence of metabolic syndrome

The prevalence rates of MS based on the three different criteria were 25.60% (NCEP ATP III), 36.68% (modified NCEP ATP III) and 19.80% (IDF criteria) (Figure 1B). Note that analysis using modified NCEP ATP III, showed a consistently higher prevalence of the MS compared to the other two criteria, namely NECP ATP III, and IDF (Figure 1B).

### Demographic and clinical profile of subjects

**Figure 2:**
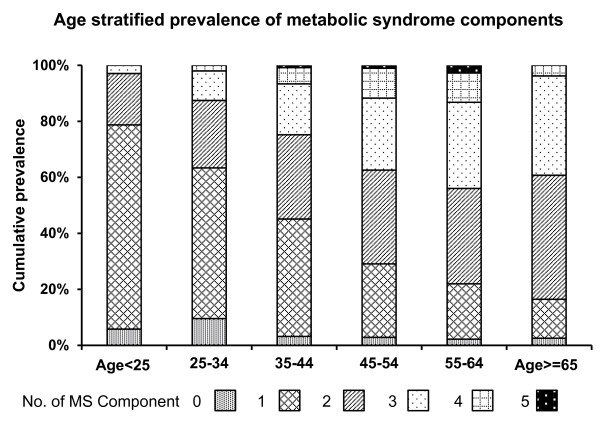
**Age stratified prevalence of metabolic syndrome (MS) components of Bangladeshi rural women (NCEP ATP III criteria, n = 1535)**. (0 = no MS component present, 1 = one component of MS present,2 = two components MS present, 3 = three components MS present, 4 = four components MS present, 5 = five components MS present).

According to the NCEP ATP III criteria, the highest prevalence of MS in rural Bangladeshi women was 43.96% for the age group between 55 to 64 years (Figure 1C). The incidence of MS was found to increase with age, e.g., 2.90% of those below 25 years had MS compared to 43.96% for the age group between 55 to 64 years (Figure 1C). However, due perhaps to the poor response by the 65 years + age group, the prevalence of MS in this age group was found to be lower than the age group between 55 to 64 years, a group that had the highest prevalence. Numbers of MS components increase with advancement in age; particularly two and three components for this population were observed to increase (Figure 2). Among the subjects, 4.56% females had no components of MS, while 0.78% females had all 5 components of MS.

Age classified MS characteristics shows that mean systolic and diastolic blood pressure, in rural Bangladeshi women were increasing with the advancement of age; mean cholesterol and fasting blood glucose appeared to increase till age group 45-54(Table 1). BMI was statistically significant for the age group 35-44 and age ≥ 65 compared to the age group < 25. WC was statistically significant for almost all age groups compared to the group age < 25; SBP and DBP were also statistically significant for all age groups compared to the group age < 25. Age dependent increase was significant for LDL for 45-54 age group compared to the group age < 25. The mean fasting blood glucose has been shown to be significant for 35-44, 45-54, 55-64 and ≥ 65 age groups compared to the group age < 25. Mean triglyceride and cholesterol levels were significant for 45-54 and 55-64 age groups compared to the group age < 25. However, there was no statistically significant age-dependency for HDL cholesterol increase.

**Table 1:** Characteristics of respondents by age and MS components

	Age < 25	25-34	35-44	45-54	55-64	Age ≥ 65	Total
**BMI (kg/m^2^)**	20.11 ± 0.26	21.26 ± 0.23	21.76 ± 0.22*	21.18 ± 0.21	21.12 ± 0.43	22.25 ± 0.61*	21.26 ± 0.11
**Waist circumference (cm)**	72.95 ± 0.62	77.95 ± 0.54*	79.02 ± 0.49*	77.53 ± 0.49*	77.44 ± 0.88*	74.54 ± 0.9	77.35 ± 0.25
**Systolic BP (mmHg)**	98.47 ± 0.98	106.18 ± 0.89*	115.36 ± 1.02*	122.09 ± 1.3*	132.35 ± 2.23*	136.29 ± 3.07*	115.62 ± 0.62
**Diastolic BP (mmHg)**	67.56 ± 0.69	71.35 ± 0.53*	75.61 ± 0.51*	77.48 ± 0.59*	81.74 ± 1.05*	84.14 ± 1.52*	75.16 ± 0.3
**Cholesterol (mg/dL)**	153.87 ± 5.51	166.58 ± 3.82	170.29 ± 3.48	189.37 ± 4.05*	187.09 ± 5.71*	168.17 ± 8.61	173.88 ± 1.89
**LDL cholesterol (mg/dL)**	97.09 ± 5.23	111.16 ± 3.61	110.46 ± 3.24	125.9 ± 3.85*	120.95 ± 5.44	110.92 ± 7.61	113.92 ± 1.77
**HDL cholesterol (mg/dL)**	33.64 ± 0.79	34.32 ± 0.67	34.87 ± 0.56	33.54 ± 0.64	33.7 ± 1.01	34.26 ± 1.71	34.13 ± 0.31
**TG (mg/dL)**	102.9 ± 5.95	93.59 ± 3.69	117.8 ± 4.52	139.02 ± 5.65*	150.66 ± 8.99*	115.32 ± 11.24	119.31 ± 2.44
**Fasting blood glucose (mmol/L)**	5.06 ± 0.06	5.39 ± 0.09	6.14 ± 0.15*	6.79 ± 0.2*	6.65 ± 0.31*	7.07 ± 0.36*	6.1 ± 0.08

Values are represented as Mean ± SE; Significance level, * *P *< 0.05

Table 2 shows the various socio-demographic and clinical parameters that have been compared between respondents with and without MS (as per NCEP ATP III criteria). We found a significant difference in MS prevalence by SES indicators. MS is more common among the respondents in higher socioeconomic status (SES) than lower SES. Higher prevalence was found among the respondents with higher family income, land ownership and larger housing space (MS vs. non-MS: family income ≥ 140 USD 5.9% vs. 2.7%, family income 70-139 USD 20.9% vs. 15.6%, family income < 70 USD 73.3% vs. 77.8%, *p *= 0.071; family having land 70.5% vs. 51.8%, family having no land 27.2% vs. 43.1%, *p *= < 0.001; family living area > 1,000 sq. ft. 83.5% vs. 70.4%, family living area ≤ 1,000 sq. ft. 16.5% vs. 29.6%, *p *= < 0.001). Educated respondents without MS were more compared to those with MS (MS vs. non-MS: having formal education 43.5% vs. 50.6%, having no education 56.5% vs. 49.4%, *p *= 0.017). Parental history of diabetes mellitus (DM) and hypertension were significantly more among those with MS than in non-MS group (MS vs. non-MS: parental history of DM 18.1% vs. 9.0%, *p *< 0.001; parental history of hypertension 23.2% vs. 15%, *p *= 0.003). However, tobacco use was not significant between MS group and non-MS group, it is rather contrasted. Current tobacco users constituted 18.8% among MS and 21.7% among non-MS.

**Table 2:** Socioeconomic status of study subjects corresponding to metabolic syndrome

Parameters	Subjects without MS (%) n = 1142		Subjects with MS (%) n = 393		*p *value
Family Income					0.071
Income < 70 USD	888	(77.8%)	288	(73.3%)	
Income 70-139 USD	178	(15.6%)	82	(20.9%)	
Income ≥ 140 USD	31	(2.7%)	23	(5.9%)	
Missing	45	(3.9%)	0	(0%)	
Own Land					< 0.001
No Land	492	(43.1%)	107	(27.2%)	
Land	591	(51.8%)	277	(70.5%)	
Missing	59	(5.2%)	9	(2.3%)	
Living Area					< 0.001
Living Area ≤ 1,000 Sqr. ft	338	(29.6%)	65	(16.5%)	
Living Area > 1,000 Sqr. ft	804	(70.4%)	328	(83.5%)	
Education Level					0.017
No Formal Education	564	(49.4%)	222	(56.5%)	
Formal Education	578	(50.6%)	171	(43.5%)	
Types of Work					0.002
Desk Work	28	(2.5%)	20	(5.1%)	
Physical Work	1060	(92.8%)	365	(92.9%)	
Missing	54	(4.7%)	8	(2.0%)	
Family History* of Diabetes					< 0.001
Non Diabetic	992	(86.9%)	322	(81.9%)	
Diabetic	103	(9.0%)	71	(18.1%)	
Missing	47	(4.1%)	0	(0%)	
Family History* of Hypertension					0.003
Non Hypertensive	924	(80.9%)	302	(76.8%)	
Hypertensive	171	(15.0%)	91	(23.2%)	
Missing	47	(4.1%)	0	(0%)	
Smoking Habit					0.242
No	894	(78.3%)	319	(81.2%)	
Yes	248	(21.7%)	74	(18.8%)	

*Family history is only accounted from father and mother only

### Prevalence of individual components of metabolic syndrome

Based on the original NCEP ATP III criteria, the most common risk factor of MS observed (Table 3) in the present study was low levels of serum HDL cholesterol, i.e., observed in 85.47% of the target population. This was immediately followed by high fasting blood glucose levels (30.42% of the target population), high blood pressure (29.12% of the target population) and elevated serum triglyceride levels (26.91% of the target population). Waist circumference levels ≥ 88 cm were found in 11.6% of individuals. When waist circumference cut-offs were reduced based on the modified NCEP ATP III criteria, low HDL was again found to be the most common risk factor followed by elevated fasting plasma glucose level and hypertension (Table 3). It is interesting to note the sharp rise in the prevalence of central obesity, when the cut-off for waist circumference is lowered to 80 cm in women (11.6% to 31.01%).

**Table 3:** Age stratified prevalence of individual components of metabolic syndrome

Age Group	Obesity Waist ≥ 88 cm**	Obesity Waist ≥ 80 cm*	High TG ≥ 150 mg/dl	Low HDL < 50 mg/dl	Fasting blood glucose ≥ 6.1 mmol/L**	Fasting blood glucose ≥ 5.6 mmol/L*	Hypertension (SBP ≥ 130 mmHg or DBP ≥ 85 mmHg)
**Age < 25**	2.90	14.98	14.01	91.79	7.73	18.36	1.93
**25-34**	14.19	40.26	17.49	87.13	16.17	29.04	6.60
**35-44**	15.83	41.16	25.59	88.92	29.55	47.23	24.01
**45-54**	11.69	29.61	35.32	84.94	43.38	56.88	42.86
**55-64**	12.09	24.18	43.96	78.02	41.76	59.89	59.89
**Age > = 65**	2.53	11.39	22.78	65.82	59.49	68.35	73.42
**All age**	11.60	31.01	26.91	85.47	30.42	44.76	29.12

Values are expressed in percentage

* for Modified NCEP ATP III and IDF criteria

** for NCEP ATP III criteria

Prevalence of individual risk factors of MS that match different age groups (as per NCEP ATP III) is given in Table 3. Highest prevalence of waist circumference (≥ 88 cm) was observed among individuals aged between 35 to 44 years (15.83%). Growing prevalence of TG ≥ 150 mg/dl was observed with advancing of age, with a difference noted in individuals aged ≥ 65 years. However, some younger people (< 25 years) were also found to have a very high prevalence of low HDL < 50 mg/dl. The prevalence of hypertension (SBP ≥ 130 mmHg or DBP ≥ 85 mmHg) and elevated fasting blood glucose ≥ 6.1 mmol/L increase with the advancement of age (Table 3).

### Risk factors for metabolic syndrome

Multivariate logistic regression analysis was used to evaluate the potential role of age, history of diabetes in parents and history of hypertension in parents in developing MS (Table 4). Advancing age emerged as one of the most significant risk factors for developing MS. Subjects in the age group 55-64 years had an odds ratio (OR) of 19.07 (CI 7.97-45.65, *p *< 0.001) as compared to those age between 15 to 24 years. Diabetes in any one of the parents (OR 2.33, CI 1.62-3.33, *p *< 0.001) was also significant independent risk factor for developing MS but hypertension in any one of the parents was not significant (OR 1.35, CI 0.99-1.84, *p *= 0.059).

**Table 4:** Multivariate logistic regression analysis for risk factors for metabolic syndrome (MS)

Risk factors for MS	Odds ratio (OR)	95% confidence interval for OR	Significance, *p*
**Age groups (in years)**	Reference group-age 15-24 years		
25-34	3.25	1.33-7.95	0.010
35-44	7.39	3.15-17.37	< 0.001
45-54	15.16	6.51-35.29	< 0.001
55-64	19.07	7.97-45.65	< 0.001
> 64	16.58	6.44-42.67	< 0.001
**Family history of DM**	Reference group-neither father or mother had DM		
Father or mother had DM	2.33	1.62-3.33	< 0.001
**Family history of HTN**	Reference group-neither father or mother had HTN		
Father or mother had HTN	1.35	0.99-1.84	0.059

## Discussion

The present study reports the first comprehensive MS epidemiological study that evaluated 1535 women of different age groups from rural Bangladesh. Based on three different criteria, the prevalence of MS was found to be 25.60% (NCEP ATP III), 36.68% (modified NCEP ATP III), and 19.80% (IDF criteria), respectively. We found a prevalence of MS that was highly age-dependent, i.e., there was an approximate four-fold increase in prevalence between age group 25-34 years to age group 55-64 years.

The prevalence of the MS in rural Bangladeshi women (36.68%), as revealed here, is similar to that reported in rural women of India (36.4%) using the NCEP ATP III criteria with ethnic specific cut off value for waist circumference [14]. Prevalence of MS was higher (36.68% vs. 25.60%) when ethnicity specific cut-offs for waist circumference were applied. Several population studies have reported an increase in the prevalence of the MS with age, regardless of definition [15-34], with some noting a peak in the seventh decade and then a decline in both sexes [15,22,26,34] or only in men [16,17,21,25,29,33]. In the present study, the prevalence of MS increased with age but a slight decline as noted after the age of 65 years, consistent with findings reported in India [35]. The prevalence of MS was the lowest in the age group below 25 years (2.90%), while it progressively increased with age, plateauing between 55 to 64 years (43.96%). These findings indicate that aging may be a risk factor for MS for Bangladeshi rural women.

In the present study, based on the original NCEP ATP III criteria, low serum HDL levels was found to be the most common risk factor of MS, .i.e., it was observed in 85% of the target population. It is important to note that mean HDL levels were also found to be lower than the normal range even in the non-metabolic group (Non-MS vs. MS; 42.48 ± 1.05, 37.03 ± 1.47). When WC cut-offs specific for Asians were used as baseline for analysis, low HDL again emerged as the most common risk factor of MS followed by elevated plasma glucose level and hypertension. This observation in the pattern of profile MS in the present study is consistent with reports of previous studies performed in India [21,36]. These Indian studies actually demonstrate a significantly higher prevalence of low HDL cholesterol in Indian women (90.2%) as compared to US women (39.3%). In the present study, a consistently high prevalence (> 80-90%) of low HDL was observed across generations, i.e., from young to old. This observation in our target population is critical considering that epidemiological studies have established a strong inverse association between HDL concentrations with increased risk of coronary artery disease. For every mg/dl decrease in HDL, the risk for CAD increases by 2-3% [37]. However, the causality of this relationship is hard to prove [38].

Low HDL can be either monogenic or purely environmental or, in most cases is multifactorial/polygenic in origin [39]. HDL levels are under considerable genetic control with heritability estimates of up to 80% [40]. Besides, the high prevalence of low HDLC even in many individuals without obesity and hypertriglycemia, suggests an ethnic predisposition to this type of dyslipidemia. Reports of disproportionately high prevalence of low HDLC in South and Middle East Asia have been accumulating recently [41]. Gupta et al. suggested that this could be due to a high prevalence of hypoalphalipoproteinemia in the Indian population and needs to be confirmed in larger prospective studies [21]. Prevalence of high TG and low HDL-C might contribute to the high prevalence rate of MS in this study population. Metabolism and the genes associated with HDL and TG are reported, at least in part, to be linked to each other [42]. Further analysis is needed to explore the influence of life style, food pattern or physical activity of Bangladeshi rural women on such type of dyslipidemia.

The prevalence of many of the components of MS has been found to be increased over age in the present study. The prevalence of elevated fasting blood glucose and hypertension increased with age in rural Bangladeshi women. The overall prevalence of hypertension in this target population was 29.43%. This hypertensive rate is higher than previously reported, i.e., prevalence of 18.2% [43]. The higher prevalence of elevated BP in our subjects may be attributed to their higher intake of saturated fat and high calorie foods. Prevalence of high blood level of TG increased, peaking between 55 to 64 years. However, in the case of HDL level there was no age-specific prevalence. These patterns in prevalence, as revealed by the present study, were similar to those in a cross-sectional population survey in urban Asian Indian adults reported by [44]. Consistent to other reports [35], we also found an age-dependent increase in the prevalence of elevated fasting plasma glucose level. Thus from the present findings, we state that age might be a strong risk factor for the high prevalence of many of the components of MS in Bangladeshi rural women although the most common prevalent component (low level of HDL) did not shown any age-specific distribution pattern.

In the present study, elevated waist circumference is the least frequent component of MS in rural Bangladeshi women based on the ATP III criteria. The average waist circumference in this population was found to be 74.80 cm, which is consistent with another study of rural Bangladeshi women which reported a waist circumference of 68 cm [43]. Based on these findings, we speculate that many Bangladeshis women are metabolically obese but physically non-obese. In the present study, even when we re-evaluated our data using the modified Asian waist circumference cut-off, we still found that 18.05% of our participants did not have central obesity, even though they still had MS. Another point noteworthy is that both urban males and females had significantly higher WC and WHR (waist hip ratio) compared with their rural counter-parts, according to an Indian study by Das. This means that urban dwellers have a significantly higher central adiposity compared with rural dwellers [14]. We do not know whether there is a similar pattern in Bangladesh. We currently have an ongoing study assessing the profile of MS in Bangladeshi urban female population.

In our study, MS was significantly more prevalent among upper SES compared to lower SES which indicates SES as an emerging risk factor of MS in developing countries like Bangladesh and this finding is consistent with a study by Deepa et al., the prevalence of MS among persons belonging to middle income group was significantly higher compared to those from lower income group (18.7% vs. 6.5%) [45]. But it should be noted that the percentage of upper SES people were few in the present study population.

Few studies have demonstrated not only increased prevalence of diabetes but also other cardio metabolic risk factors including glucose intolerance and insulin resistance [46], central obesity, abnormal triglyceride and HDL among offspring of diabetic parents [47]. In our study, those with history of diabetes in either or both parents had significantly increased risk of having metabolic syndrome and other cardio metabolic risk factors including central obesity, abnormal triglyceride and HDL. Also, those whose both parents were diabetic were significantly more at risk than if only one of their parents had diabetes, thus showing evidence for genetic predisposition for developing metabolic syndrome and cardio metabolic risk factors. Similar result has been observed in a study done in 321 adolescent Indians [48]. In that study, the study subjects whose both parents had diabetes had significantly more prevalence of cardio metabolic risk factors as compared to those whose parents did not have diabetes. Thus genetic factors may play a potential role for MS development in Bangladeshi population.

The present report complements earlier MS reports performed in Bangladesh [7,49]. The study by Rahim was also conducted in a rural community, but in the outskirts of Dhaka city. The criteria they used to assess the prevalence of MS were modified ATP vs. IDF vs. WHO and their findings based on these criteria were 25.1% vs. 15.7 vs. 9.2%, respectively. The figures obtained in their study were lower compared to the present study. Discrepancies in such studies are a common. For instance, the prevalence of MS in different parts of India varied from 11% to 41% [5,36,50]. The underlying causes for the observed differences between studies within the same region and country may be attributed to various factors, including the type of criteria employed (EGIR, ATP III and IDF), target age groups and variation in the pattern or sets of risk factors of MS. Bhopal's findings that a large proportion (65%) of women in Bangladesh had low HDL cholesterol level, are consistent with those of the present study [51].

In the present study, as we determined the prevalence of metabolic syndrome in Bangladeshi rural women, so we have difficulty in including the parameter work type (desk work type or physical work type) to our multivariate logistic regression analysis model. In current investigation, 45 (3.03% of total study participants) subjects were categorized to desk work and the most of the subjects fell into physical work categories (1345, 93.27% of total study participants) and some missing cases (55 subjects, 3.7% of total subjects). More intensive investigation is warranted in future in this respect. Through a cross sectional study design it is not possible to make a concrete interpretation at this regard.

The present manuscript shows MS is an important public health problem in Bangladesh although very few studies have been done on it. The high prevalence of MS may also have serious implications for health care costs in rural Bangladesh. Thus, studies designed to examine the direct medical costs associated with MS are urgently needed.

Several steps can be taken to reduce the prevalence of potential CVD risk factors in this population. A potential initiative for dietary modifications and enhanced physical activity may have implications to reduce this public health burden [52-54]. While proper management of the individual risk factors and abnormalities of MS can reduce morbidity and mortality, a more integrated strategy will provide a better outcome. Implementation of the strategies cited here will require a community outreach. Thus, education and training of health care providers is critical. It is important to ensure that health care providers have the knowledge and skills necessary to not only treat MS patients but organized an effective MS prevention program to the community.

## Conclusions

In conclusion, the present study reveals a high prevalence of MS in rural Bangladeshi women, with low serum HDL levels as the most common risk factor. These findings should prove useful in the formulation of health policies and prevention programs.

## Abbreviations

MS: Metabolic syndrome; CVD: Cardiovascular diseases; T2DM: Type 2 diabetes mellitus; BMI: Body mass index; WC: Waist circumference; HRT: Hormone replacement therapy; IHD: Ischemic heart disease; SES: Socioeconomic status; SPSS: Statistical Package for Social Sciences; WHR: waist hip ratio.

## Competing interests

The authors declare that they have no competing interests.

## Authors' contributions

SJ developed the idea for this paper; RI, SM, SI, NS and SZ performed all searches and compiled the text, SJ contributed to the writing and NY, YI, MH and TW provided conceptual and editorial input. All authors read and approved the final manuscript.

## Pre-publication history

The pre-publication history for this paper can be accessed here:

http://www.biomedcentral.com/1471-2458/12/49/prepub
